# Benign Recurrent Aseptic Meningitis (Mollaret’s Meningitis) in an Elderly Male: A Case Report

**DOI:** 10.31729/jnma.6950

**Published:** 2021-09-30

**Authors:** Swati Chand, Sangharsha Thapa, Kushal Gautam, Anu Radha Twayana, Maryrose R. Laguio-Vila, Tarek Elsourbagy

**Affiliations:** 1 Department of Internal Medicine, Rochester General Hospital, Rochester, USA; 2 Kathmandu University School of Medical Sciences, Dhulikhel, Kavrepalanchowk, Nepal; 3 Department of Pediatric Research, Patan Academy of Health Sciences, Lalitpur, Nepal; 4 Department of Internal Medicine, Rochester General Hospital, Rochester, USA; 5 Faculty of Medicine, Cairo University, Egypt

**Keywords:** *aseptic meningitis*, *benign recurrent*, *Mollaret's meningitis*

## Abstract

Mollaret's meningitis is an aseptic recurrent benign lymphocytic meningitis lasting 2-5 days and occurs over years with spontaneous complete resolution of symptoms between episodes. An 88 years-old-male presented with acute onset headache, lethargy and altered sensorium after a recent ear infection. He had multiple similar episodes in the past, each preceded by ear or sinus infection with cerebrospinal fluid finding consistent with aseptic meningitis. However, no specific causative agent was ever identified. He was confused, disoriented and lethargic with normal vitals and systemic examination. Blood tests showed leukocytosis with neutrophilia. Cerebrospinal fluid analysis revealed increased cell count with lymphocyte predominance, elevated protein and negative polymerase chain reaction. Magnetic resonance imaging of brain showed chronic small vessel ischemic changes. He fulfilled the Bruyn's criteria for clinical diagnosis. He was empirically administered acyclovir during hospitalization and was discharged without prophylactic antiviral due to negative cerebrospinal fluid analysis, culture and multiplex polymerase chain reaction.

## INTRODUCTION

Mollaret's meningitis is an aseptic lymphocytic meningitis occurring over a period of several years and characterized by greater than three episodes of meningitis symptoms lasting 2-5 days with spontaneous and complete resolution of symptoms between the episodes.^1^ Neurological findings like mental status change, seizures, hallucinations, coma and cranial nerve palsies can be associated.^2^ It is a rare disease with over 100 cases reported in the literature, resulting in cases being missed or overly investigated and treated.^3^ We present a case of Mollaret's meningitis in an 88 years old male who presented with altered mental status after a recent ear infection.

## CASE REPORT

An 88-year-old male presented with headache, lethargy and altered sensorium. Three days prior to presentation, he was assessed for earache and started on amoxicillin. Since then, he gradually became lethargic, drowsy and acutely confused. He did not have fever, light sensitivity, neck stiffness, nausea, vomiting, head trauma, myalgia, arthralgia, gastrointestinal upset and no history of genital ulcer. He denied recent travel, insect bite, animal contact and history of similar symptoms in the family or in the vicinity.

His past medical history included 5 previous similar episodes requiring hospitalization over 10 years. Each hospitalization was followed by extensive evaluation with documented aseptic lymphocytic meningitis without specific etiology. Each episode was preceded by ear or sinus infection and lasted 5-10 days with complete resolution without residual neurological symptoms. He was previously on prophylactic antiviral therapy for a year that was discontinued 3 years prior to this episode. Other medical conditions included coronary artery disease, hypertension, hyperlipidemia, and gastro esophageal reflux disease.

On examination, the patient was lethargic and confused. His pulse rate was 88 bpm, blood pressure 147/69 mm Hg, temperature 37.7 °C, respiratory rate 18 per minute, and oxygen saturation 97%. Physical examination was remarkable for altered sensorium (inattentive, disoriented, non-communicative) without any focal neurological deficit. There was no lymphadenopathy or rashes. Cardiac, pulmonary, abdominal and genital examinations were normal. White cell count was elevated to 16,600 WBCs per microliter with neutrophil predominance (84%). Electrolytes, urine and chest x-ray were normal. Urine toxicology was negative. Non-contrast Computed tomography (CT) head showed no acute infarct or hemorrhage. Magnetic resonance imaging (MRI) of brain showed mild to moderate chronic small vessel ischemic changes. Electroencephalogram showed intermittent bursts of slowing in the right frontal region suggestive of focal neurological dysfunction in the right frontotemporal region. Cerebrospinal fluid (CSF) was clear with a cell count of 139 with lymphocytes of 82%, protein 122 mg/dl and glucose 46mg/dl. CSF fluid was negative for syphilis by Venereal Disease Research Laboratory test (VDRL), and multiplex PCR testing for Herpes simplex virus-1 (HSV-1), Herpes simplex virus-2 (HSV-2), Varicella zoster virus (VZV), Cytomegalovirus, Enterovirus, Human parechovirus, Escherichia coli, Haemophilus influenzae, Listeria monocytogenes, Neisseria meningitidis, Streptococcus agalactiae, Streptococcus pneumoniae, Cryptococcus neoformans were negative. CSF bacterial culture and blood culture were negative.

The patient initially received antibiotics empirically which was discontinued on day 2. He was then managed with acyclovir for 10 days. He improved and was discharged on day 10. On discharge, he had no neurological deficit and no prophylactic antiviral was started.

## DISCUSSION

Mollaret's meningitis, a rare entity, is characterized by recurrent episodes of acute lymphocytic meningitis with symptom free period and normal cerebrospinal fluid analysis in between the episodes. Several conditions like para-meningeal infections (sinusitis, mastoiditis), post traumatic bacterial meningitis, and certain drugs are recognized as a preceding cause for recurrent meningitis.^4^ This patient presented with features suggestive of meningitis preceded by ear or sinus infection in each episode.

Mollaret's Meningitis was first described by Pierre Mollaret, French neurologist in 1944 as a form of recurrent aseptic meningitis without identified specific cause.^4^ Large endothelial-like cells, known as Mollaret cells (Ghost cells) are noted in the CSF analysis within 12-24 hours but they are not considered pathognomonic.^2,5^ With advanced diagnostic modalities; currently different viruses have been associated with it. HSV-2 has been identified as the most common causative agent and treatment involves use of antiviral drugs. After primary infection, HSV-2 remains dormant within the sensory neurons of dorsal root ganglia and retrograde seeding of the virus can lead to recurrent meningitis.^6^ However, alternative causes such as other viruses (HSV-1, VZV, EBV) and non-infectious etiologies such as Central nervous system (CNS) epidermoid cyst, craniopharyngioma have been identified. ^2,7,8^ Herpes simplex viruses have unique biologic and pathogenic properties that help establish recurrent human infections. HSV has the capacity to invade and replicate in the CNS and the capacity to establish latent infection in dorsal root ganglia in Peripheral Nervous System.^9^ Following entry, HSV infects nerve endings and translocate by retrograde transport to the nuclei of sensory ganglia.^6^ Seeding of the virus from the ganglia can lead to recurrent meningitis. Recurrences are spontaneous and mostly triggered by factors like physical or emotional stress, fever and immune imbalance.^2^

Bruyn's criteria were described in 1962 to make the diagnosis of Mollaret's meningitis (Table 1).^10-11^

**Table 1:** Bruyn's Criteria for the clinical diagnosis of Mollaret's meningitis.

Attacks separated by symptom free period of weeks to months Spontaneous remission of symptoms and signs Recurrent episodes of severe headache, meningismus and fever CSF pleocytosis with large endothelial cells, neutrophils and lymphocytes No causative etiological agent has been identified

This patient fulfilled the criteria for the diagnosis. Patient had recurrent clinical presentation and CSF profile consistent with aseptic meningitis followed by resolution with symptom free interval. No causative agent was identified during any episode.

Given that many cases of Mollaret's meningitis have reported HSV-2 as the cause, Bruyn's criteria may not be applicable at present. Some authors have advised restricting the term Mollaret's meningitis to cases with no etiology, like the case we presented. Since HSV had never been identified, prophylactic antivirals was not started. Patient has not had another episode in the following 4 months (until now).

Mollaret's meningitis usually resolves after a period of 3-5 years, but cases with longer periods and more episodes, similar to this patient have also been reported.^4,11^ This was his 6^th^ episode in a course of 10 years. Given the rarity of diagnosis of this syndrome it is difficult to perform any study or trials on it. Sudden onset of symptoms with its presentation indistinguishable from other forms of life-threatening meningitis and episodes that occur over course of years pose a challenge to physicians to make an accurate diagnosis early.^4^ Consequently, patients often undergo repeated hospitalizations, lengthy work ups and empiric management with antimicrobials. HSV-2 has been more commonly identified in cases with this diagnosis now.^5,9^ However, there are no studies to show efficacy of antivirals over symptomatic management or importance of prophylactic treatment with antivirals.^2^

It is important that physicians be aware and vigilant about this condition to prevent unnecessary investigations, exposure to empiric antimicrobials and prolonged hospital stay with associated considerable cost.
